# Current advances of bone homeostasis imbalance in the cause of hereditary metabolic bone diseases

**DOI:** 10.1530/EOR-2025-0147

**Published:** 2026-02-04

**Authors:** Xinyu Dai, Yiwei Wang, Xuanhe Huang, Zhanyu Meng, Pengfei Zheng

**Affiliations:** Department of Orthopaedics Surgery, Children’s Hospital of Nanjing Medical University, Nanjing, Jiangsu Province, China

**Keywords:** bone homeostasis, osteoblast, osteoclast, osteogenesis imperfecta, Paget’s disease of bone, hypophosphatemic rickets, osteopetrosis

## Abstract

- Bone homeostasis, a dynamic equilibrium essential for skeletal development and repair, is coordinately regulated by osteoclasts, osteoblasts, and osteocytes.
- Hereditary metabolic bone diseases arise from genetic mutations that impair the function of these key bone cells, disrupting the homeostatic balance.
- This review specifically addresses four prevalent hereditary metabolic bone diseases: osteogenesis imperfecta, Paget’s disease of bone, hypophosphatemic rickets, and osteopetrosis.
- Dysfunction in major signaling pathways – notably the Wnt/β-catenin, RANK/RANKL/OPG, and TGF-β/BMP pathways – plays a central role in the aberrant bone remodeling underlying these disorders.
- Elucidating the molecular mechanisms involving these cells and pathways is fundamental to understanding disease pathogenesis and is crucial for the development of novel therapeutic interventions, presenting significant promise for future research.

## Introduction

The skeletal system is the most basic supporting structure of the human body, which not only provides mechanical support and protection but also serves as an important reservoir for calcium and participates in a variety of physiological processes. The normal operation of the skeletal system is inseparable from the stability of bone homeostasis. Bone homeostasis is the process by which bone tissue maintains a dynamic balance of structure and function during bone development and repair. This complex process involves three major types of bone cells: osteoclasts, osteoblasts, and osteocytes (1). They act synergistically to maintain bone homeostasis through the regulation of a variety of signaling molecules and pathways. In bone homeostasis, the balance between osteoblasts and osteoclasts constitutes the main body of bone homeostasis, and their effects are closely related (2). Currently, it is known that the imbalance of bone homeostasis directly affects bone health, leading to a variety of bone diseases. For example, excessive osteoclast activity can lead to diseases such as Paget’s disease of bone (3). Similarly, a diminished action of osteoblasts may trigger osteomalacia or rickets (4).

Hereditary metabolic bone diseases comprise one of the most diverse groups among rare diseases. These disorders are characterized by genetic abnormalities in bone homeostasis that may lead to abnormal calcium transport, phosphate content, and vitamin D metabolites (5). Hereditary metabolic bone diseases can be classified into disorders of defective bone mineralization, disorder of bone matrix and cartilage formation, and sclerosing bone disorders (6). Disorders of defective bone mineralization are characterized by defective mineralization of newly synthesized organic bone, leading to reduced mechanical strength and bone deformability (7). Disorders of bone matrix and cartilage formation originate from abnormalities in the synthesis or structure of the organic bone matrix. Even with normal mineralization processes, defects in the quality or quantity of the bone matrix compromise structural integrity, leading to increased bone fragility and susceptibility to fracture (8). In contrast, the pathogenesis of sclerosing bone disorders is relatively distinct. The core abnormality lies in severe dysregulation of bone remodeling – specifically, impaired osteoclast function leading to reduced bone resorption – resulting in high bone density but poor structural integrity and increased fracture risk (9).

It is essential to provide early and accurate diagnosis and treatment for patients with a hereditary metabolic bone disease to maintain bone development and improve quality of life and to prevent fractures and other metabolic complications. This review gives an overview of the classification and characteristics of four representative hereditary metabolic bone diseases and discusses the mechanism of bone homeostasis imbalance in the process of inherited metabolic bone diseases, revealing its key role in the development of these diseases.

## Mechanisms of bone homeostasis

### Bone remodeling process

The bone remodeling cycle consists of five stages, activation, resorption, reversal, formation, and termination, and occurs over several weeks (10). In each bone area undergoing remodeling, cells involved in bone resorption and formation gather in temporary structures called ‘basal multicellular units’ (BMUs) (11). Each BMU is encased by bone-lining cells. The active BMU consists of osteoclasts that cover the exposed bone surface in preparation for dissolution of the bone. Osteoblasts appear after osteoclasts and secrete and deposit unmineralized bone osteoid.

Bone is in a quiescent state before bone remodeling begins. During the activation phase, the bone detects the initiating remodeling signal. This signal can be hormonal, such as estrogen or parathyroid hormone, or mechanical strain (12). After the signal is detected, the endosteal membrane is digested by the action of collagenase. The second step is involved in recruitment and dissemination of osteoclast progenitors. Then, the large multinucleated osteoclasts migrate and attach to the bone surface. During bone resorption, osteoclasts secrete hydrogen ions and enzymes digest bone matrix. Multinucleated osteoclasts undergo apoptosis at the end of this phase (10). Currently, although the coupling signals between bone resorption and formation have not been fully elucidated, a number of coupling factors have been identified over recent years. These factors include the following: i) proteins released from the resorbed bone matrix, ii) proteins secreted by osteoclasts, iii) membrane-bound proteins presented on the osteoclast surface, and iv) exosome-associated proteins and miRNAs released by osteoclasts (13). Ma *et al.* found that osteoclast-derived apoptotic bodies promote osteogenic differentiation of osteoblastic stem cells via RANKL reverse signaling, demonstrating that apoptotic bodies play an important biological role in coupling bone formation with resorption during bone remodeling (14). Osteoblasts synthesize the proteinaceous new matrix to fill the cavities left by osteoclasts. As the new bone matrix is gradually mineralized, new bone is gradually formed. After completion of bone formation, 50–70% of osteoblasts undergo apoptosis, and the rest become osteocytes or bone-lining cells (10).

### Major signaling pathways that regulate bone homeostasis

#### Wnt signaling pathway

The Wnt signaling pathway is an important cell signal transduction pathway, which plays a key role in embryonic development, cell proliferation, differentiation, and tissue homeostasis. The Wnt signaling pathway is primarily categorized into two types: the classical Wnt/β-catenin pathway and the non-classical Wnt signaling pathways, encompassing the Wnt/Ca^2+^ pathway and the Wnt–planar cell polarity pathway (15).

In the classical Wnt/β-catenin pathway, Wnt ligands bind to Frizzled receptors and activate low-density lipoprotein receptor-related protein 5 or 6 (LRP5/6) as a co-receptor. This binding inhibits the degradation of β-catenin, resulting in its accumulation in the cytoplasm and eventual translocation to the nucleus to regulate the transcription of target genes (16).

**Figure 1:**
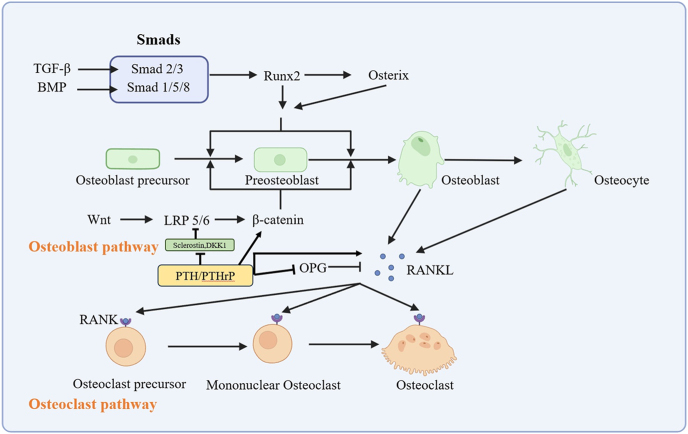
Common signaling pathways regulating osteoblast and osteoclast differentiation. Osteoblast pathway: TGF-β signaling and BMP signaling activate distinct Smad complexes (Smad2/3 and Smad1/5/8, respectively), which promote osteoblast differentiation via transcriptional factors Runx2 and Osterix. The Wnt/β-catenin pathway (through LRP5/6 receptors) synergizes with Smad signaling to enhance osteoblast maturation. Osteoclast pathway: RANKL binds to RANK on osteoclast precursors, triggering osteoclast differentiation. OPG acts as a decoy receptor to inhibit RANKL–RANK signal. Mononuclear osteoclasts fuse to form multinucleated mature osteoclasts responsible for bone resorption. PTH/PTHrP signaling regulates bone homeostasis through crosstalk with key pathways. Sustained PTH increases RANKL and decreases OPG in osteoblasts, promoting bone loss. Intermittent PTH enhances bone formation by downregulating Wnt inhibitors (sclerostin and DKK1) and elevating β-catenin. PTH also stimulates BMP-2/4 expression, activating BMP signaling to promote osteogenesis.

The non-classical Wnt signaling pathway is not dependent on the accumulation of β-catenin. An increasing amount of evidence indicates the role of the non-classical Wnt signal in the crosstalk between bone and the pathway. The study by Tu *et al.* suggests that Wnt7b promotes the differentiation of osteoblasts via PKCδ signaling (17). WNT5a also exerts a role in osteoblastogenesis by inhibiting PPAR-γ transcription to suppress adipogenesis, involving the inactivation of chromatin (18) (Fig. 1).

#### RANKL/RANK/OPG pathway

RANKL, a ligand secreted by osteoblasts, binds to the RANK on the surface of osteoclasts, facilitating the generation and activation of osteoclasts. The expression of RANKL can be modulated by multiple factors, including hormones, mechanical loads, and inflammatory cytokines (19).

RANK is a receptor situated on the surface of osteoclasts and their precursor cells. After RANKL binds to RANK, it activates a series of signal transduction pathways, including the NF-κB pathway, thereby facilitating the development, fusion, and functional activation of osteoclasts. This process intensifies bone resorption and results in bone mass loss.

OPG is a soluble protein generated by osteoblasts and other cell types, such as endothelial cells and macrophages. OPG acts as a ‘decoy receptor’ of RANK. By binding to RANKL, it thereby precludes the interaction between RANKL and RANK, inhibits the formation and function of osteoclasts, and slows down the process of bone resorption (20). Under various pathological conditions (such as osteoarthritis and osteoporosis), overexpression of RANKL or a decrease in OPG may cause an increase in bone resorption, thereby triggering bone loss and the occurrence of bone diseases (Fig. 1).

#### TGF-β and BMP signaling pathway

TGF-β and BMP transduce signals to both Smad-dependent signaling pathway and non-Smad-dependent signaling pathway to regulate bone formation and bone homeostasis. The members of the TGF-β family mainly consist of TGF-β1, TGF-β2, and TGF-β3. TGF-β initiates its cellular responses by binding to a tetrameric receptor complex consisting of two TGF-β type I receptors (TβRI) and two type II kinase receptors (TβRII). In the complex, TβRII transphosphorylates TβRI, resulting in the phosphorylation of receptor-activated Smads (R-Smads), which mainly include Smad2/3. R-Smads subsequently interact with common Smad (Co-Smad), mainly Smad4, and are translocated to the nucleus, where they recruit co-factors to regulate gene transcription. Bosch *et al.* indicated that TGF-β can also activate another group of R-Smads (Smad1/5/8) through binding to activin receptor-like kinase-1 (ALK1) (21). Furthermore, a non-Smad-dependent pathway exists, where kinase 1 (TAK1) and TAK1-binding protein 1 (TAB1) can trigger MKK3–p38 MAPK or ERK–MAPK signal cascades (22).

BMP family encompasses 14 proteins, among which BMP-2, BMP-4, BMP-5, BMP-6, BMP-7, and BMP-9 demonstrate a relatively high osteogenic activity (23). The osteogenic capabilities of BMP-2 and BMP-7 have been widely investigated. BMP-2 significantly increased the expression of osteocalcin, and short-term expression of BMP-2 was necessary for inducing bone formation (24). In contrast, BMP-7 elicits the expression of osteogenic differentiation markers (such as alkaline phosphatase) and expedites bone mineralization (25).

The BMP signaling pathway is mediated via type I and type II BMP receptors. When the BMP ligand binds to the type II receptor, the type II receptor forms a tetrameric complex with the type I receptor. In the classical pathway, BMP signaling proceeds via Smad1/5/8 as its R-Smad. Similar to TGF-β, the Smad1/5/8–Smad4 complex transcribes the runt-related transcription factor 2 (Runx2) gene and complexes with Runx2 to initiate the expression of other osteogenic genes (26). The non-classical pathway of BMP signaling also participates in the regulation of osteogenic differentiation, and the TAK1–MKK–MAPK pathway plays an important role in the regulation of BMP effects (27).

The regulatory mechanism of the BMP signaling pathway is complex, and the activity of BMP is antagonized by binding proteins such as Noggin (28), Grem1 (29), and Chordin (30). I-Smad (inhibitory Smad), including Smad6 and Smad7, inhibits BMP and TGF-β signal in multiple ways. Smad7 binds TGF-β type I receptor blocking R-Smad phosphorylation and eventually TGF-β signaling (31). The chondrocyte transgenic mice specifically expressing Smad6 give rise to osteogenic dysfunction by inhibiting the Smad1/5/8 signaling pathway (32) (Fig. 1).

#### PTH and PTHrP signaling pathway

Parathyroid hormone (PTH) and its related peptide PTHrP regulate extracellular calcium and phosphate metabolism and control bone growth and repair via their common receptor, type 1 PTH/PTHrP receptor (PTH1R) (33). PTH is a polypeptide hormone secreted by the parathyroid glands, mainly acting on bones and kidneys to regulate the metabolism of calcium and phosphorus. PTHrP, conversely, is synthesized by multiple tissues, including skin, blood vessels, and smooth muscle (34).

PTH and PTHrP induce a series of intracellular signal transduction responses by activating PTH1R. The signal transduction of PTH1R predominantly depends on heterotrimeric G proteins, such as G_s_, G_q/11_, G_i_, and G_12_/G_1_ (35). At present, there exist signal transduction modalities that do not rely on G proteins, for instance, the mitotic signal transduction of extracellular signal-regulated kinases 1 and 2 (ERK1/ERK2) (36).

In the process of bone remodeling, PTH stimulates bone formation by facilitating the differentiation of osteoblast precursors and suppressing the apoptosis of mature osteoblasts and osteocytes (37). PTHrP is regarded as relatively independent in promoting bone formation from the effect of PTH in promoting bone formation (38). The regulation of bone homeostasis by the PTH/PTHrP signaling pathway is largely achieved through complex crosstalk with other key signaling pathways. PTH and PTHrP act on osteoblasts, where PTH stimulates the gene expression and protein secretion of RANKL while simultaneously suppressing the mRNA expression of OPG in osteoblasts (39). This constitutes the primary mechanism through which sustained high levels of PTH lead to bone loss. In contrast, intermittent administration of PTH promotes bone formation (40). PTH can enhance osteogenesis by downregulating Wnt pathway inhibitors, such as sclerostin and dickkopf-1 (DKK1), and by increasing the expression of β-catenin (41). In addition, the pro-osteogenic effects of PTH can be mediated through the stimulation of BMP-2/4 expression and subsequent activation of the BMP signaling pathway (42) (Fig. 1).

## Pathogenic mechanisms of bone homeostasis imbalance in some common hereditary metabolic bone diseases

### Osteogenesis imperfecta (OI)

#### Clinicopathological characteristics and classification systems

OI is a rare hereditary disorder of skeletal development, whose clinical features mainly include fragile bones, growth retardation, and short stature. In 1979, Silent *et al.* classified OI into four types based on clinical characteristics and the severity of the disease. Type I OI: the quality of collagen is normal, but the quantity is insufficient. It is mild and accompanied by blue sclera. Type II OI: also referred to as the neonatal lethal type or congenital OI, both the quality and quantity of collagen are insufficient. It is lethal and accompanied by blue sclera. Type III OI: the quantity of collagen is sufficient, but the quality is poor. It is severe, and the color of the sclera is variable. Type IV OI: it is moderately severe with normal sclera (43). Furthermore, there are numerous other rarer types (Type V to Type XXI), which are induced by mutations in distinct genes (44).

#### Molecular mechanisms of imbalance in bone homeostasis

In OI, the function of osteoblasts is compromised, resulting in the deterioration of the quality of bone matrix. Meanwhile, the activity of osteoclasts escalates, leading to intensified bone resorption. Such an imbalance of bone homeostasis gives rise to increased bone fragility and an elevated risk of fractures. The majority of OI are attributed to the structural or quantitative defects of the COL1A1 or COL1A2 genes encoding the α1(I) and α2(I) chains of type I collagen, resulting in the obstruction of type I collagen synthesis. The synthesis of collagen is a complex process. The collagen I α1 and collagen I α2 chains synthesized in the endoplasmic reticulum undergo extensive modifications by chaperone proteins. Once reaching the Golgi apparatus, the procollagen is transported to the extracellular matrix, where collagen fibers are formed (45). The study by Mirigian *et al.* indicated that in the pathological condition of OI, active osteoblasts generate a significant amount of collagen. The accumulation of these misfolded procollagens in the endoplasmic reticulum leads to an unconventional form of cellular stress, which is neither the traditional unfolded protein response stress nor endoplasmic reticulum overload (46). Gorrell *et al.* conducted a study on how osteogenesis responds to the accumulation of misfolded procollagen in the endoplasmic reticulum and how this response influences the function of osteoblasts. The research discovered that, in the G610C mouse model, the misfolded procollagen evaded the quality control of the endoplasmic reticulum lumen and initiated an integrated stress response (ISR) through contact with other cell compartments. The activation of the ISR causes osteoblasts to reduce protein synthesis in response to the disruption of the normal protein folding and transport processes caused by the accumulation of procollagen. The activation process of the ISR is modulated by mitochondrial HSP70 (mt-HSP70) and activating transcription factor 5 (ATF5). mt-HSP70 is a heat shock protein in the mitochondria, indicating that the mitochondria are associated with the initiation of the ISR (47).

The activation of the TGF-β/Smad signaling pathway is the most significant dysregulated event in the bones of children with OI, and the phosphorylation level of Smad proteins rises significantly (48). Gebken *et al.* indicated that the number of TGF-β receptors on the cell surface of adult osteoblasts in OI was higher than that of the matched controls (49). The research conducted by Shi *et al.* indicated that the caveolae-mediated endocytosis and degradation of TβRI were diminished in mice, simultaneously accompanied by a reduction in the phosphorylation of caveolin-1 (Cav-1). Type I collagen binds to integrin α2β1 to function, activating the phosphorylation of downstream focal adhesion kinase (FAK) and Src and thereby causing the phosphorylation of Cav-1. However, in OI, the synthesis of type I collagen is blocked and the phosphorylation of Cav-1 is decreased. This results in an increase in the TGF-β receptors on the cell membrane surface and an upregulation of the TGF-β/Smad signaling pathway (50).

Furthermore, the Wnt1 mutations in patients with OI can cause fragile bones. Osteocytes constitute the main source of Wnt1. Joeng *et al.* suggested that Wnt1 in osteocytes activates the mTORC1 pathway, facilitating the proliferation and mineralization of osteoblasts (51). Mesenchymal cells are also capable of secreting Wnt1. Wnt1 is expressed on the membrane surface of cells, and osteogenic precursor cells combine with Wnt1 via the Frizzled receptor on the cell membrane, resulting in the nuclear translocation of β-catenin. This contact-dependent signal transduction requires a mode of juxtacrine signaling. The Wnt1 signal can also decrease osteoclast formation by inhibiting the expression of RANKL and increasing its inhibitors (such as sclerostin and DKK1) to maintain the balance of bone mass (52). This response leads to a reduction in the mineralized surface and the rate of bone formation. The number of osteoclasts and the surface for bone resorption increase.

Type V OI is caused by a heterozygous mutation (c, −14 C > T) in the 5′-untranslated region of the interferon-induced transmembrane protein 5 gene (IFITM5). IFITM5 encodes the bone-restricted ifitm-like protein (BRIL), a transmembrane protein enriched during the mineralization process of osteoblasts. Its specific function remains unclear. Although it does not directly involve in the formation of type I collagen, the hyper-mineralization of the bone matrix has an impact on collagen formation. The BRIL mutant activates transcription factors such as MEF2, NFATc, and NR4A significantly in osteoblasts (53). The mutated IFITM5 activates the ERK signaling and the downstream SOX9 protein, which is the master regulator for cartilage development. This mutation can suppress the differentiation of chondrogenic cells into osteoblastic cells, resulting in excessive cartilage growth and defects in bone formation (54).

Type VI OI is caused by a null mutation in SERPINF1, which encodes the pigment epithelium-derived factor (PEDF). It is characterized by the accumulation of unmineralized osteoid and the fish-scale pattern of bone lamellae. The deficiency of PEDF affects osteocyte differentiation and mineralization. PEDF and TGF-β show antagonistic effects during osteogenesis and vascularization. PEDF inhibits the expression of pro-angiogenic factors induced by TGF-β, while TGF-β suppresses the expression of osteogenic marker genes. The absence of PEDF causes the pathological characteristics of type VI OI by enhancing TGF-β signaling and promoting bone vascularization. In conclusion, the deficiency of PEDF gives rise to the pathological characteristics of type VI OI by enhancing TGF-β signaling and facilitating bone vascularization (55).

The deficiency of cartilage-associated protein (CRTAP) can result in an extremely rare autosomal recessive type VII OI. The biallelic mutations of c.621 + 1G > A and c.1153 − 3C > G in CRTAP can cause a decrease in CRTAP mRNA in osteoblasts and a deficiency of CRTAP protein, thereby reducing the 3-hydroxylation of Pro986 in the α1 chain of type I collagen. Consequently, the volume of osteoid cells and the number of osteoblasts are significantly decreased, resulting in severe type VII OI (56).

Type XII OI results from a loss-of-function mutation in SP7. Osterix encoded by SP7 is a zinc finger-structured transcription factor and an indispensable component involved in bone formation. It can restrain the proliferation of immature osteoblasts, induce the maturation of osteoblasts and the expression of Col1a1, and is necessary for the proliferation process of bone cells, which can prevent high cortical porosity (57). Osterix is a crucial regulatory factor for osteoblast differentiation and acts at the early stage of bone formation. It functions downstream of Runx2 and promotes the maturation of osteoblasts and the deposition of bone matrix (58, 59).

Type XIV OI is a recessive type of OI caused by a null mutation in TMEM38B. The endoplasmic reticulum membrane TRIC-B encoded by TMEM38 transports K^+^ ions. Jovanovic *et al.* demonstrated via RNA-Seq analysis that the cell–cell adhesion-related pathway is one of the most downregulated pathways in TMEM38B-null osteoblasts. The impairment of gap and tight junction proteins results in the obstruction of cell proliferation and cell cycle progression. In addition, it was discovered that the mitochondria in osteoblasts were elongated, and the generation of superoxide within mitochondria was significantly augmented (60). Besio *et al.* found at the cellular level that the delayed differentiation of osteoblasts in type XIV OI and the obstruction of collagen synthesis were also associated with Ca^2+^ imbalance. The alteration of the Ca^2+^ calmodulin kinase II (CaMKII)-mediated signaling pathway led to a decrease in Smad phosphorylation and nuclear translocation (61).

Furthermore, in OI, alterations in the immune system also influence the equilibrium of bone homeostasis. Kang and his team discovered that T cells in mice with OI displayed activated phenotypes and secreted elevated levels of pro-inflammatory cytokines (such as IFN-γ and TNF-α). Through systemic transplantation of regulatory T cells (Tregs), the activation state of T cells can be diminished and the secretion of pro-inflammatory factors can be decreased. After the transplantation, the parameters of trabecular and cortical bones in OI mice were significantly ameliorated, and the mechanical strength of the bone was enhanced. In *in vitro* culture experiments, Tregs can inhibit osteoclast formation and promote the mineralization of osteoblasts. This indicates that autoimmune therapy might potentially be employed for the treatment of OI in the future (62) (Table 1).

**Table 1:** Common OI genetic classification and unique features.

OI type	Defective gene	Protein	Homeostasis imbalance
I–IV	*COL1A1* or *COL1A2*	Collagen I a1 or a2	Upregulation of the TGF-β/Smad signaling pathway
V	*IFITM5*	BRIL	Activation of ERK signaling and downstream SOX9 protein
VI	*SERPINF1*	PEDF	Enhancing TGF-β signaling and promoting bone vascularization
VII	*CRTAP*	CRTAP	3-Hydroxylation of Pro986 in the α1 chain of type I collagen, leading to the decrease in the volume of osteoid cells and the number of osteoblasts
XII	*SP7*	Osterix	Deficiency of osterix, a downstream target of Runx2, resulting in impaired osteoblast differentiation
XIV	*TMEM38B*	TRIC-B	Downregulation of cell–cell adhesion and the alteration of the CaMKII-mediated signaling pathway, leading to a decrease in Smad phosphorylation and nuclear translocation

### Paget’s disease of bone (PDB)

#### Clinicopathological characteristics and classification systems

The prominent characteristic of Paget’s disease of bone is an exceptionally active bone turnover and disordered bone remodeling. Among the population, the incidence rate of Paget’s disease of bone is approximately 1–2%, among which the main affected population is aged over 55 years (63). In 15–40% of cases, the genetic mode of Paget’s disease is autosomal dominant. Mutations in the *SQSTM1* gene are the most common genetic cause of this disease. Analyses of different populations have indicated that the most prevalent mutation type is pP392L. The bone biopsy specimens of Paget’s disease of bone reveal a disordered bone matrix with poor structural stability, chaotic collagen fibers, and a considerable number of abnormal osteoclasts (6).

#### Molecular mechanisms of imbalance in bone homeostasis

The major abnormalities in PDB exist in osteoclasts, where the size of osteoclasts and the number of multinucleated cells increase compared with normal osteoclasts. The differentiation and function of osteoclasts rely on the interactions among three molecules: RANK encoded by TNFRSF11A, RANKL encoded by TNFSF1, and OPG encoded by TNFRSF11B. RANK receptors are expressed by osteoclasts and osteoclast precursors; RANKL is produced by osteocytes, activated T cells, osteoblasts, and bone marrow stromal cells, and OPG is produced by osteoblasts and other cell types. The binding of RANKL to RANK promotes osteoclast differentiation and bone resorption.

In PDB, the augmented sensitivity of osteoclast precursors to RANKL results in increased bone resorption (64). Miyagawa *et al.* utilized measles virus nucleocapsid protein (MVNP) transgenic mice and TRAP-Igf1 transgenic mice through immunohistochemistry and molecular biology techniques (65). They analyzed the expression levels of sclerostin and RANKL in bone cells of mice with different genotypes, as well as the morphological characteristics of bone cells. They discovered that insulin-like growth factor-1 (IGF-1) from osteoclasts plays a crucial role in the pathogenesis of PDB. The increase in IGF-1 induces bone cell senescence and promotes the generation of RANKL in bone cells, thereby inducing the formation of lesions. IGF-1 not only promotes the production of RANKL through osteocyte but also possibly enhances the formation and activity of osteoclasts, leading to more IGF-1 secretion and forming a positive feedback mechanism (3).

Optineurin (OPTN) is encoded by the OPTN gene in humans and by the Optn gene in mice. The Optn-knockout mice display the late-stage symptoms of PDB. In PDB associated with Optn, the activity of osteoblasts was significantly enhanced, and the gene expression of the TGF-β/BMP signaling pathway regulating osteoblast differentiation was upregulated. Hu *et al.* indicated that the secretions of osteoclasts could restore the osteogenic induction capacity in OPTN-deficient mice and restore the function of osteoblasts (66). Mice deficient in Optn also exhibited a marked increase in osteoclastogenesis. Wong *et al.* discovered that OPTN deficiency results in dual defects in the production of IFN-β and IFN-α/βR signal transduction. This defect precludes the effective upregulation of anti-osteoclast factors, thereby leading to excessive activation of osteoclasts and the occurrence of osteolytic lesions (67). Xue *et al.* also identified another pathway that causes osteoclast abnormalities. OPTN directly interacts with NRF2 in the cytoplasm. A reduction in the expression of NRF2 and its related antioxidants subsequently leads to abnormal osteoclasts. Moreover, the use of NRF2 activators (such as curcumin) can mitigate the excessive osteoblastogenesis caused by OPTN deficiency (68).

About 70% of patients with Paget’s disease of bone have osteoclasts that express MVNP, which induces high expression of interleukin-6 (IL-6) by downregulating the Foxo3/Sirt1 signal (69). The high expression of IL-6 simultaneously augmented the expression of IGF-1, thereby increasing the formation of osteoclasts and bone damage. Teramachi *et al.* discovered that IL-6 enhanced the expression of coupling factors, particularly EphrinB2 in osteoclasts and EphB4 in osteoblasts. Furthermore, the EphrinB2–EphB4 coupling was strengthened, and bone formation was enhanced (70). Sambandam *et al.* discovered that the expression of NFAM1 in the bone marrow cells of patients with PDB was significantly elevated, and MVNP was capable of further upregulating the level of NFAM1. MVNP influences intracellular calcium levels by modulating NFAM1, thereby facilitating the differentiation of osteoclasts. NFAM1 also governs the activity of signaling molecules such as Syk, PLCγ, and calmodulin, thereby influencing the expression and nuclear translocation of NFATc1. This suggests that MVNP enhances the formation of osteoclast and bone resorption activity in PDB patients through the NFAM1 signaling pathway, potentially offering a novel perspective for understanding the pathological mechanism of PDB (71).

**Figure 2:**
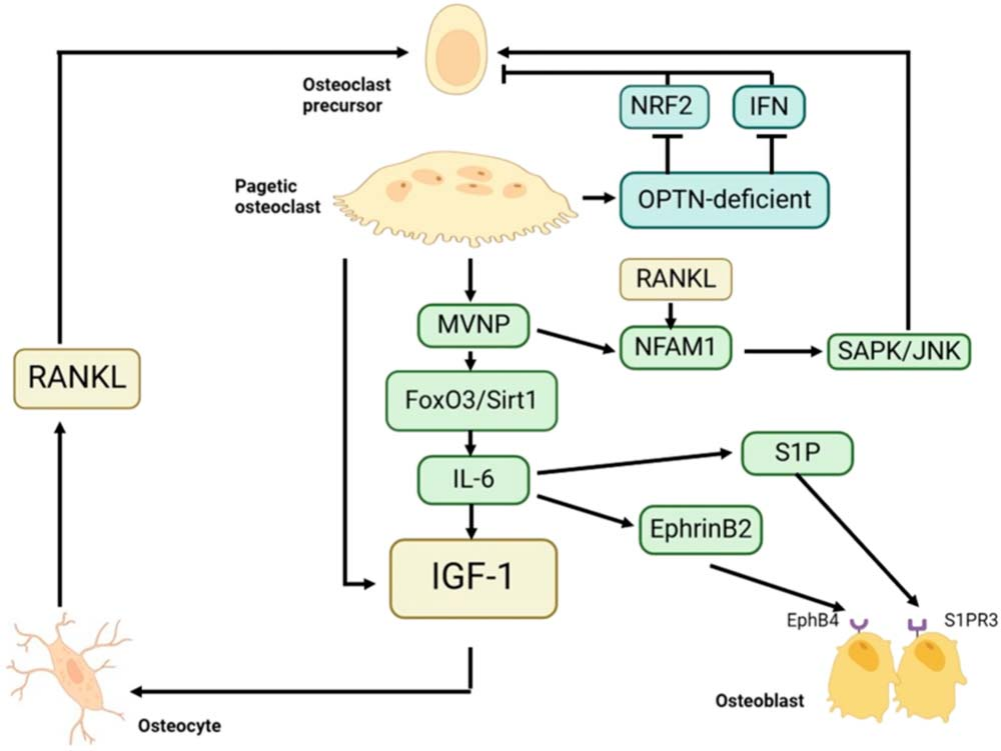
Dysregulated bone homeostasis in Paget’s disease of bone (PDB). This schematic illustrates the molecular and cellular mechanisms underlying aberrant osteoclast–osteoblast coupling in PDB. Key factors include the activation of the RANKL signaling pathway due to increased IGF-1 levels. Activated osteoclasts secrete more IGF-1, forming a positive feedback loop. Genetic factors such as OPTN deficiency and MVNP expression may drive pathological bone remodeling. OPTN deficiency induces dual defects in the NRF2 and IFN pathways, further impairing osteoclast function. MVNP expression downregulates FoxO3/Sirt1 signaling, thereby inducing elevated IL-6 production, which in turn promotes IGF-1 expression. IL-6 also enhanced the expression of coupling factors, particularly EphrinB2 in osteoclasts and EphB4 in osteoblasts. Furthermore, the EphrinB2–EphB4 coupling was strengthened. Meanwhile, S1P released by osteoclasts promotes osteoblast differentiation and bone formation by binding to S1PR3 on osteoblasts. MVNP-induced activation of NFAM1 and SAPK/JNK pathways further promotes osteoclast activation.

However, MVNP is not the sole reason for the abnormal levels of NFAM1. The research conducted by Ethiraj *et al.* revealed that the aberrant increase in RANKL in PDB can enhance the expression of NFAM1 in precursor osteoclasts. NFAM1 governs osteoclast differentiation and bone resorption by regulating the SAPK/JNK signaling pathway (72). Nagata *et al.* through the analysis of osteoclasts in PDB patients and MVNP mice discovered that sphingosine-1-phosphate (S1P) released by osteoclasts promotes osteoblast differentiation and bone formation by binding to S1P-receptor-3 (S1PR3) on osteoblasts. The IL-6 produced by osteoclasts in PDB patients and MVNP mice can upregulate the expression of S1P and S1PR3 (73) (Fig. 2).

### Hypophosphatemic rickets (HR)

#### Clinicopathological characteristics and classification systems

Hypophosphatemic rickets is a hereditary disease, characterized by bone mineralization defects resulting from hypophosphatemia and accompanied by excessive circulation of fibroblast growth factor 23 (FGF23). X-linked dominant hypophosphatemic rickets (XLDHR) is the most prevalent type of hypophosphatemic rickets, and it is caused by a loss-of-function mutation of the PHEX gene on the X chromosome. Autosomal dominant hypophosphatemic rickets (ADHR) and autosomal recessive hypophosphatemic rickets type 1 and 2 (ARHR1 and ARHR2) are relatively rare. ADHR is induced by a mutation of the FGF23 gene at its enzymatic cleavage site, which hinders the degradation of FGF23. ARHR1 and ARHR2 are, respectively, related to mutations in DMP1 and ENPP1, both of which indirectly result in an elevated level of FGF23 in circulation (6).

#### Molecular mechanisms of imbalance in bone homeostasis

XLHR is elicited by a loss-of-function mutation of the PHEX gene on the X chromosome. PHEX is expressed in osteoblast cell lines and is expressed at a higher level in osteocytes (4). The low-mineralization lesions surrounding osteocytes are a distinctive characteristic of the bones in XLHR patients, suggesting an abnormal osteocyte function. The exact physiological role of PHEX and the mechanism by which its absence leads to elevated FGF23 levels remain unknown. Yuan *et al.* utilized the Cre recombinase driven by the osteocalcin promoter to conditionally eliminate the Phex gene in osteoblasts and osteocytes, resulting in a phenotype that was nearly identical to that of Hyp mice, indicating that impaired PHEX function in osteoblasts and/or bone cells alone is sufficient to trigger the disease (74).

At present, the research on the pathogenic mechanism of the PHEX gene is rather insufficient. Miyagawa *et al.* discovered that the enhanced FGFR signal in osteoblasts of PHEX-deficient might lead to the overproduction of FGF23 by comparing the gene expression between hypophosphorous mice and wild-type control mice (75). Matrix extracellular phosphoglycoprotein (MEPE) belongs to the small integrin-binding ligand, N-linked glycoproteins (SIBLING) family. Rowe *et al.* discovered that PHEX binds to MEPE through the acidic serine-aspartate-rich MEPE-associated motif (ASARM) located in the C-terminal region of MEPE. When PHEX is dysfunctional, MEPE is not protected, resulting in excessive degradation and the release of a large quantity of ASARM peptides. The increase in ASARM peptides directly inhibits mineralization, causing insufficient mineralization in bone tissue and explaining why the loss of functional PHEX expressed by osteoblasts leads to mineralization defects in HYP (76).

Farrow *et al.* in their animal studies discovered that in ADHR mice under a low-iron diet, the Fgf23 mRNA in bone tissue increased significantly. ADHR mice presented obvious osteomalacia under a low-iron diet, with a high proportion of bone matrix surface and increased bone matrix thickness demonstrated in bone tissue. *In vitro* experiments revealed that iron deficiency upregulated the expression of Fgf23 in osteoblasts by activating hypoxia-inducible factor 1α (77).

ARHR1 is caused by an inactivating mutation of the DMP1 gene, which encodes an extracellular matrix protein belonging to the SIBLING family. Martin *et al.* revealed that the excessive production of FGF23 in Dmp1-deficient mice is attributed to the activation of the FGFR pathway (78). Through the specific deletion of FGF23 in osteoblasts and osteocytes, Li *et al.* discovered that the reduction of FGF23 could completely rectify hypophosphatemia but only partially ameliorate bone growth and mineralization. This indicates that the deficiency of DMP1 directly impacts the mineralization of osteoblasts (79).

ARHR2 is caused by an inactivating mutation in the ENPP1 gene, which encodes an ectoenzyme that catalyzes the hydrolysis of ATP into AMP and inorganic pyrophosphate (PPi). Currently, the mechanism by which the deficiency of ENPP1 leads to excessive production of FGF23 remains undefined (Table 2).

**Table 2:** Genetic causes of hypophosphatemic rickets.

Disease	Gene	Protein	Inheritance	Homeostasis imbalance
X-linked dominant hypophosphatemic rickets	*PHEX*	Phosphate-regulating endopeptidase	X-linked dominant	Enhanced FGFR signal in osteoblasts of PHEX-deficient might lead to the overproduction of FGF23
Autosomal dominant hypophosphatemic rickets	*FGF23*	Fibroblast growth factor 23	AD	Activation of hypoxia-inducible factor upregulated the expression of *FGF23* in osteoblasts
Autosomal recessive hypophosphatemic rickets type 1	*DMP1*	Dentin matrix acidic phosphoprotein	AR	Enhanced FGFR signal in osteoblasts
Autosomal recessive hypophosphatemic rickets type 2	*ENPP1*	Ectonucleotide pyrophosphatase	AR	Unclear

### Osteopetrosis (OP)

#### Clinicopathological characteristics and classification systems

Osteopetrosis is a rare hereditary metabolic bone disorder characterized by a dysfunction or absence of osteoclasts, which results in a defect in bone resorption. This gives rise to severe clinical manifestations, including increased bone density, the absence of bone marrow cavities, growth retardation, macrocephaly, progressive deafness, blindness, hepatosplenomegaly, and severe anemia.

According to the mode of inheritance, osteopetrosis can be categorized into autosomal recessive osteopetrosis (ARO), autosomal dominant osteopetrosis (ADO), and X-linked osteopetrosis (XLO). Both ARO and XLO have poor clinical prognoses, while ADO, which is also referred to as the adult benign type, has two subtypes (80).

#### Molecular mechanisms of imbalance in bone homeostasis

ARO is caused by gene mutations associated with the function or differentiation of osteoclasts. Gene mutations related to the function give rise to osteoclast-rich osteopetrosis, while those related to the differentiation lead to osteoclast-poor osteopetrosis (81).

In osteoclast-rich osteopetrosis, the most common cause is a mutation in the TCIRG1 gene. This mutation leads to more than 50% of ARO cases. TCIRG1 encodes the a3 subunit of the vacuolar-type H^+^-ATPase, which is involved in the acidification of the osteoclast resorptive cleft and transport of lysosomes for secretion (82). In ARO, the absence of TCIRG1 expression in parietal cells of the stomach impairs the reduction of gastric pH and decreases the uptake of Ca^2+^ in the intestine. Consequently, the occurrence of some ARO cases is accompanied by rickets. The TCIRG1 gene also encodes the N-terminal-truncated isoform TIRC7, which is a transmembrane protein that is almost exclusively expressed in immune tissues (83). Zhang *et al.* discovered through the study of TCIRG1 knockdown in bone marrow-derived monocytes that the volume of osteoclasts differentiated therefrom was significantly decreased. In osteoclasts with TCIRG1 knockout, the expression and nuclear translocation of NFATc1 were diminished, which influenced the expression of genes related to osteoclast differentiation and further inhibited the function of osteoclasts. The expression of IP3R2 was also reduced in osteoclasts with Tcirg1 knockdown, resulting in a decrease in intracellular calcium oscillations. This reduction in calcium signaling restricted the nuclear translocation of NFATc1 (84).

Second, it is caused by CLCN7, accounting for 17% of ARO cases. *CLCN7* encodes H (+)/Cl (−) exchange transporter 7 (CLC-7). CLC-7 is located at the ruffled border membrane of osteoclasts, which is formed by the fusion of lysosomes with the cell membrane. CLC-7, as a chloride/proton exchange protein, is responsible for transporting chloride ions in the endogenous acidic environment of osteoclasts. Variants may lead to a decrease in its exchange function, thereby influencing the acidification capacity of osteoclasts and subsequently affecting bone dissolution and resorption (85). There are also some relatively rare genetic mutations, such as OSTM1, PLEKHM1, SNX10, and CA2, that can also cause OP (82).

The osteoclast-poor osteopetrosis is extremely rare in humans and is caused by gene mutations in *TNFSF11*, which encodes RANKL, or in TNFRSF11A, which encodes RANK (86). The *SLC29A3* gene encodes a lysosomal nucleotide transporter that is highly expressed in myeloid cells and influences osteoclast differentiation. Mutations of this gene can also lead to the osteoclast-poor osteopetrosis.

ADO is mainly classified into two subtypes, namely ADO1 and ADO2. ADO1 is attributed to the activating mutations of the LRP5 gene, resulting in enhanced bone formation by osteoblasts. Khrystoforova *et al.* conducted research in zebrafish models and discovered that the genes related to osteoclast differentiation and function (such as *acp5a*, *mmp9*, and *mmp13a*) were significantly upregulated in LRP5-deficient zebrafish (87). LRP5 mediates the Wnt signaling pathway, influencing the development and maintenance of the skeleton. This signaling pathway is of vital importance for the proliferation and differentiation of osteoclasts. ADO2 is induced by gene mutations accountable for osteoclast formation or function, with the most prevalent one being the mutation of CLCN7 (88). Both ARO and ADO2 are related to pathogenic variations of the CLCN7 gene. ARO usually involves variations in both alleles, whereas ADO2 is associated with variations in a single allele (85).

## Application directions

Based on an in-depth understanding of the molecular mechanisms of the disease, future treatment strategies should be more individualized and targeted. Abnormal cellular activities exist in hereditary metabolic bone diseases. The development of small molecule drugs or biological agents targeting specific bone homeostasis signaling pathways will become a research hotspot in the future. The cellular activities can be improved by regulating the pathways. Excessive TGF-β signaling is accompanied in OI (48). Sun *et al.* effectively reduced the fracture incidence in mice with OI by employing irisin to counteract the TGF-β/Smad signaling pathway (89). DKK1 serves as a typical inhibitor of the Wnt signaling pathway and plays a crucial role (90). Ko *et al.* discovered that by blocking DKK1, the transcription of osteogenic genes, such as osteocalcin, runx2, and osterix, in bone marrow stromal cells from patients with OI was enhanced. Simultaneously, OPG was downregulated and RANKL was downregulated, and osteoclast function was inhibited. This proved the potential of DKK1 blockade for the treatment of OI (91). Furthermore, the advancement of gene therapy technologies might offer novel approaches for directly correcting the pathogenic genes or regulating their expressions. Maurizi *et al.* employed a silicon–lipid hybrid nanoparticle to deliver small interfering RNA designed for human CLCN7, not only relieving the symptoms but also ensuring safety (92).

## Conclusion and outlook

This article reviews the mechanisms of bone homeostasis imbalance in the occurrence of hereditary metabolic bone diseases, uncovering its crucial role in disease development. The maintenance of bone homeostasis involves complex cellular and molecular interactions, and its imbalance is a significant factor contributing to hereditary metabolic bone diseases. Genetic mutations directly influence the functions of bone cells, such as the activities of osteoblasts and osteoclasts, thereby affecting the bone remodeling process and the quality of bone. The research on bone homeostasis not only contributes to understanding the fundamental pathological mechanisms of bone diseases but also offers the potential for developing novel treatment strategies. Nevertheless, despite certain advancements, current treatment approaches remain confined to symptomatic treatment rather than fundamental disease reversal.

Future studies should explore in greater depth how specific genetic variations affect the behaviors of the three types of cells in bone homeostasis and how these variations influence the molecular mechanisms of bone formation and bone resorption. The research of mechanisms is inseparable from the establishment of disease models. How to create more precise models of hereditary metabolic bone diseases by using gene editing tools will be the key point of future research. The application of human induced pluripotent stem cells (iPSCs) technology will contribute to generating bone cells or bone progenitor cells directly from patient cells, providing models that are closer to the human physiological state for the research of disease mechanisms and drug screening. Deyle *et al.* isolated mesenchymal cells from patients with OI and induced them to become iPSCs. This verified the feasibility of generating potential experimental cells from patients with genetic diseases through the combination of gene targeting and iPSC-derived technologies (93).

## ICMJE Statement of Interest

The authors declare that there is no conflict of interest that could be perceived as prejudicing the impartiality of the work reported.

## Funding Statement

This work did not receive any specific grant from any funding agency in the public, commercial, or not-for-profit sector.

## Declaration of generative AI in scientific writing

The authors declare that no generative artificial intelligence (AI) or AI-assisted technologies were used in the writing process of this manuscript. All content was solely produced by the human authors, who take full responsibility for the accuracy and integrity of the work.
